# A Rare Case of Pulmonary Mucosa-Associated Lymphoid Tissue Lymphoma Transforming into Diffuse Large B-Cell Lymphoma

**DOI:** 10.7759/cureus.1373

**Published:** 2017-06-20

**Authors:** Fasil Tiruneh, Ahmad Awan, Raka Amin, Samina Afreen, Abdullahi Musa, Wayne Davis

**Affiliations:** 1 Department of Internal Medicine, Howard University Hospital; 2 Endocrinology Fellow, UPMC Presbyterian; 3 Pulmonary Critical Care, Howard University Hospital

**Keywords:** diffuse large b-cell lymphoma, maltoma, baltoma, high grade b-cell lymphoma

## Abstract

Mucosa-associated lymphoid tissue lymphoma (MALToma) is a low grade B-cell lymphoma that develops from the lungs, intestinal tract, salivary gland, and other organs and is included under extranodal marginal zone lymphoma. When a primary pulmonary MALToma develops from bronchus-associated lymphoid tissue (BALT), it is called BALT lymphoma (BALToma). The etiology of MALToma is not clear; however, an association between chronic inflammatory conditions and BALToma has been observed. Transformation of MALToma to high grade lymphoma is very rare. We experienced a case of MALToma that had developed from the lungs in a patient who was undergoing treatment for latent tuberculosis and rapidly transformed into high grade B-cell lymphoma.

## Introduction

The incidence of mucosa-associated lymphoid tissue lymphoma (MALToma) that develops from the lungs is rare, occurring in about 0.1% of pulmonary tumors [1]. Primary pulmonary lymphomas are rare and about 70% are bronchus-associated lymphoid tissue lymphoma (BALTomas) [2]. However, the incidence of secondary lymphomas involving the lung is 25–40% [3]. Among the primary lymphomas of the lung, MALToma is the most common despite being very rare. This low-grade tumor arises from the mucosa-associated lymphoid tissue of bronchus. An association between chronic inflammatory conditions and BALToma has been observed.

## Case presentation

The patient is a 63-year-old man with a past medical history of MALToma of the lung who presented with generalized weakness for the past one month associated with cough, fever, night sweats, and shortness of breath. The patient also had anorexia and weight loss of 15 pounds over one month. He denied a history of smoking. The patient was under treatment with isoniazid and pyridoxine for latent tuberculosis based on a positive skin tuberculin test.

On physical examination, his blood pressure was 110/59 mmHg, heart rate 118 beats per minute (bpm), respiratory rate 18/min, saturating 99% on room air with a temperature of 98.9^o^F. The patient was in no cardiopulmonary distress. A cardiovascular examination revealed no jugular venous distension, normal S1-S2, and no murmurs. On lung auscultation, the patient had rales at the left lung base.

Laboratory data showed sodium 134 mEq/L, potassium 4.2 mEq/L, chloride 103 mEq/L, bicarbonate 25 mEq/L, blood urea nitrogen 17 mg/dl, creatinine 0.9 mg/dl, glucose 96 mg/dl, hemoglobin 6.6 g/dl, hematocrit 20.1%, white blood cell count 5.5 x 10^3^/microliter, and platelets 128 x 10^3^/microliter. The coagulation profile showed prothrombin time (PT) 17.2 sec, partial thromboplastin time (PTT) 44.3 sec, and international normalized ratio (INR) 1.38.

**Figure 1:**
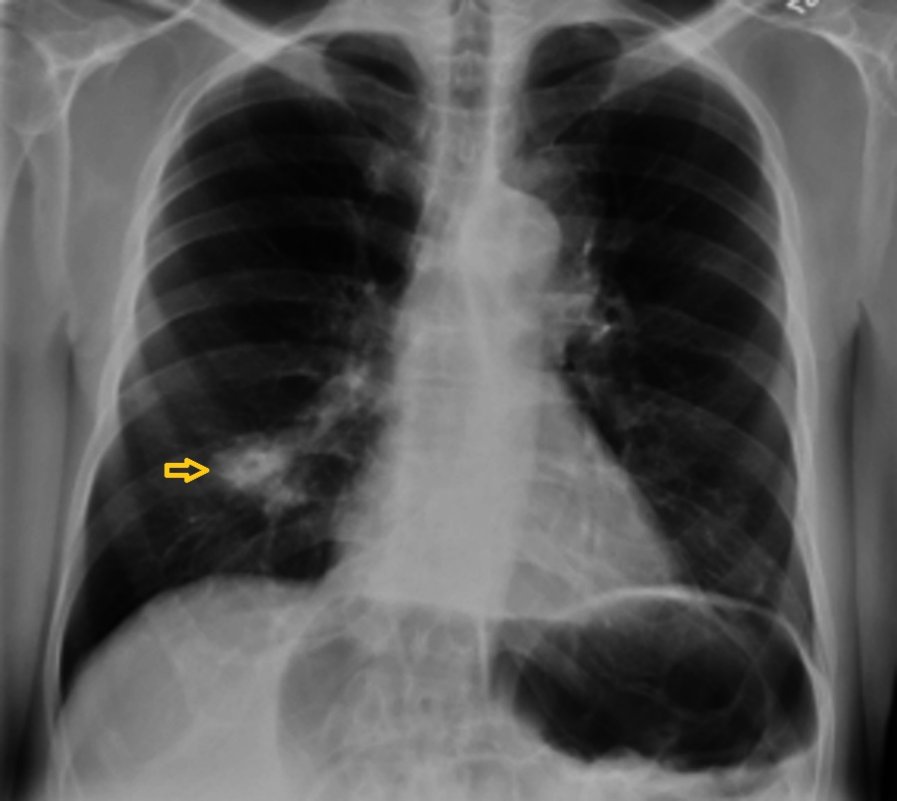
Chest X-ray showing right middle lobe opacity (arrow).

**Figure 2:**
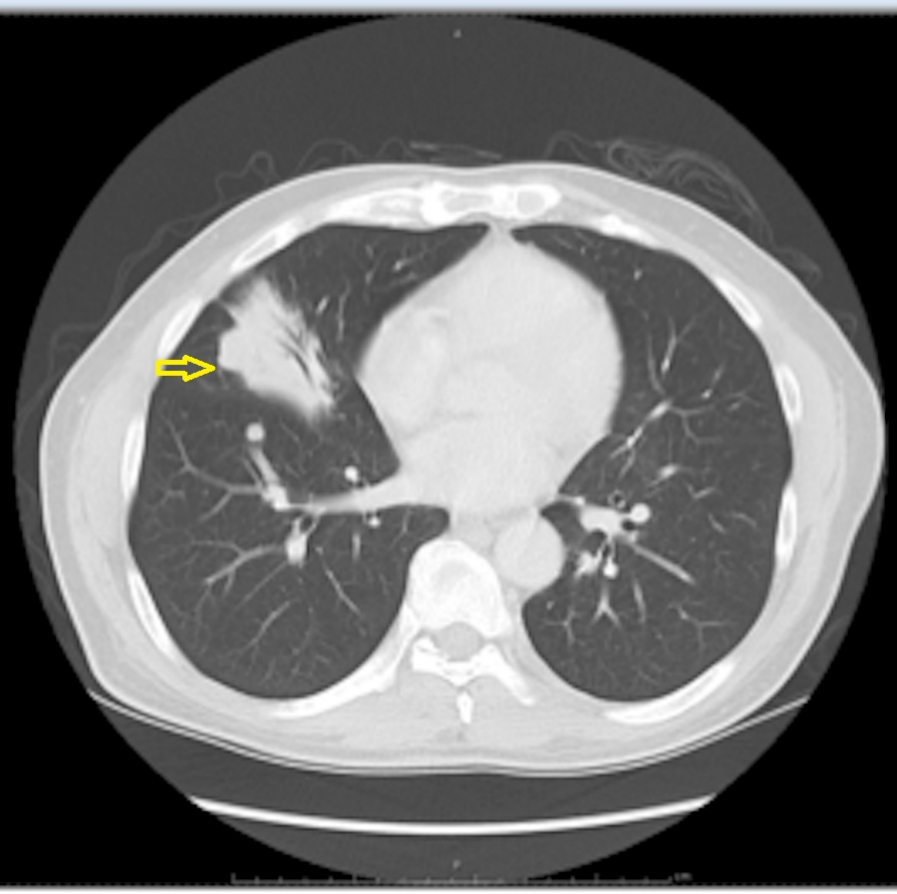
Computerized tomography (CT) of the chest showing right middle lobe opacity (arrow).

**Figure 3:**
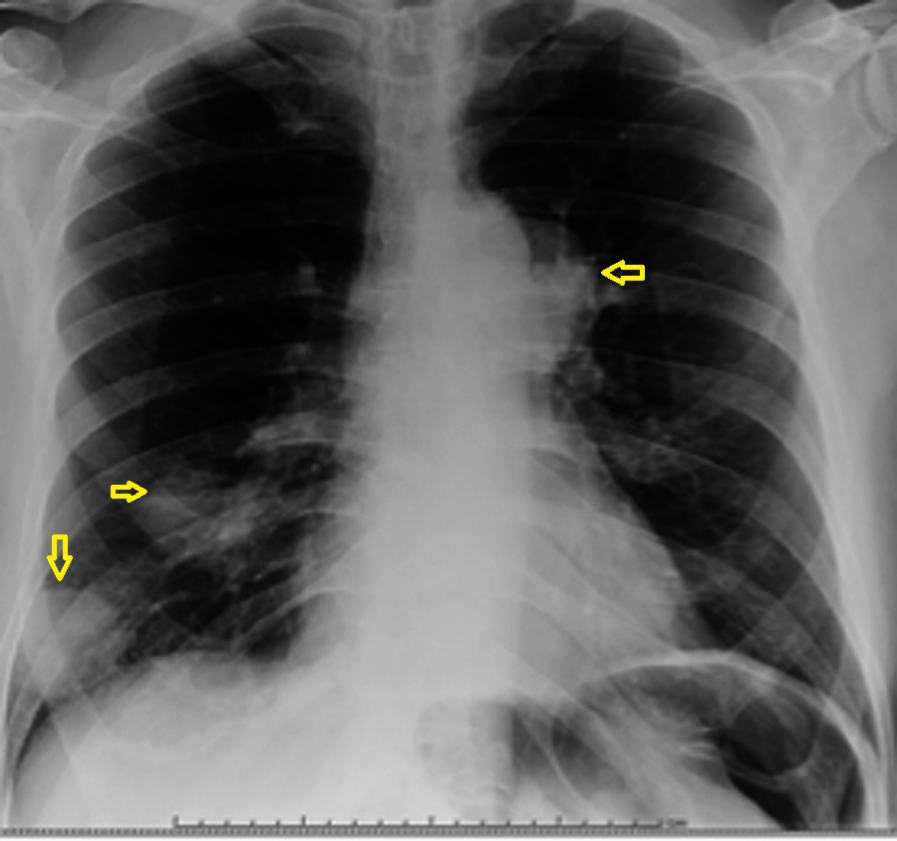
Chest X-ray showing multiple masses in the lung (arrows).

**Figure 4:**
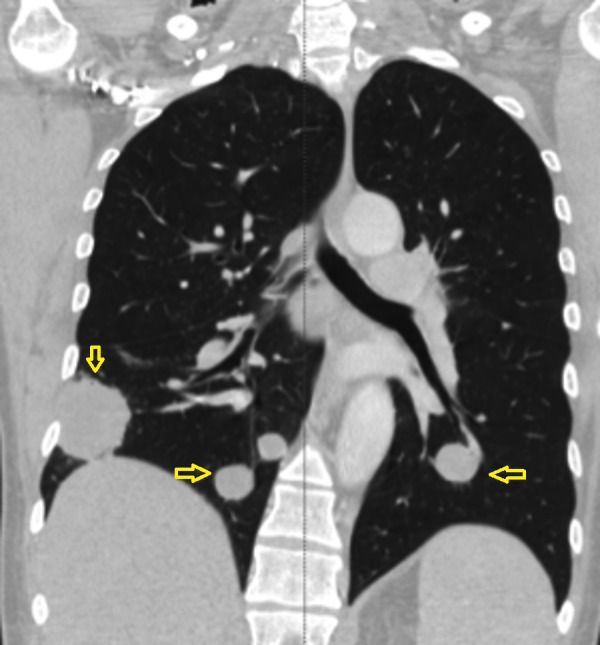
Coronal reconstructive CT scan of the chest showing multiple masses in the lung (arrows). CT - computed tomography.

**Figure 5:**
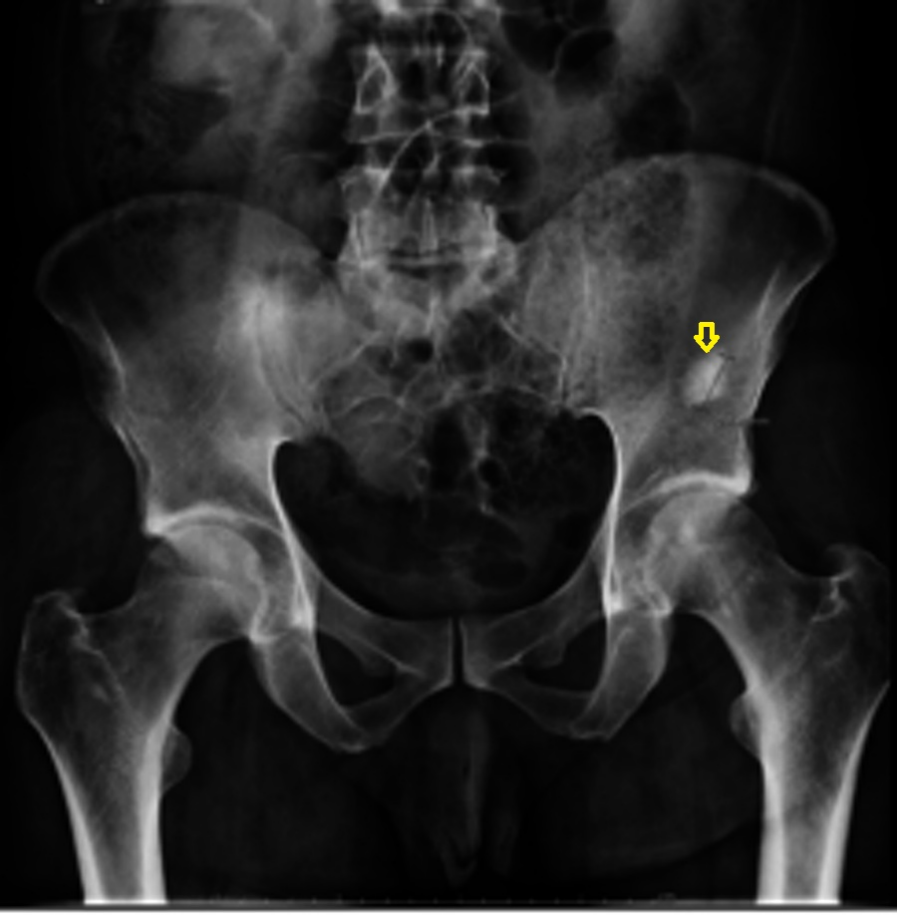
Hip X-ray showing osteoblastic lesion involving the left iliac bone (arrow).

Chest X-ray (CXR) and computed tomography (CT) scan showed right middle lobe opacity (Figures 1-2). Repeat imaging after six months showed multiple masses in the lung and an osteoblastic bone lesion involving the left ilium (Figures 3-5). Esophagogastroduodenoscopy showed a normal gastro-esophageal junction with erythematous mucosa in the stomach. The gastric biopsy was normal.

A lung biopsy done at initial presentation from the right middle lobe mass showed dense lymphoid infiltrate with proliferation of monocytoid B lymphocytes consistent with extranodal marginal zone lymphoma (BALToma/MALToma).

**Figure 6:**
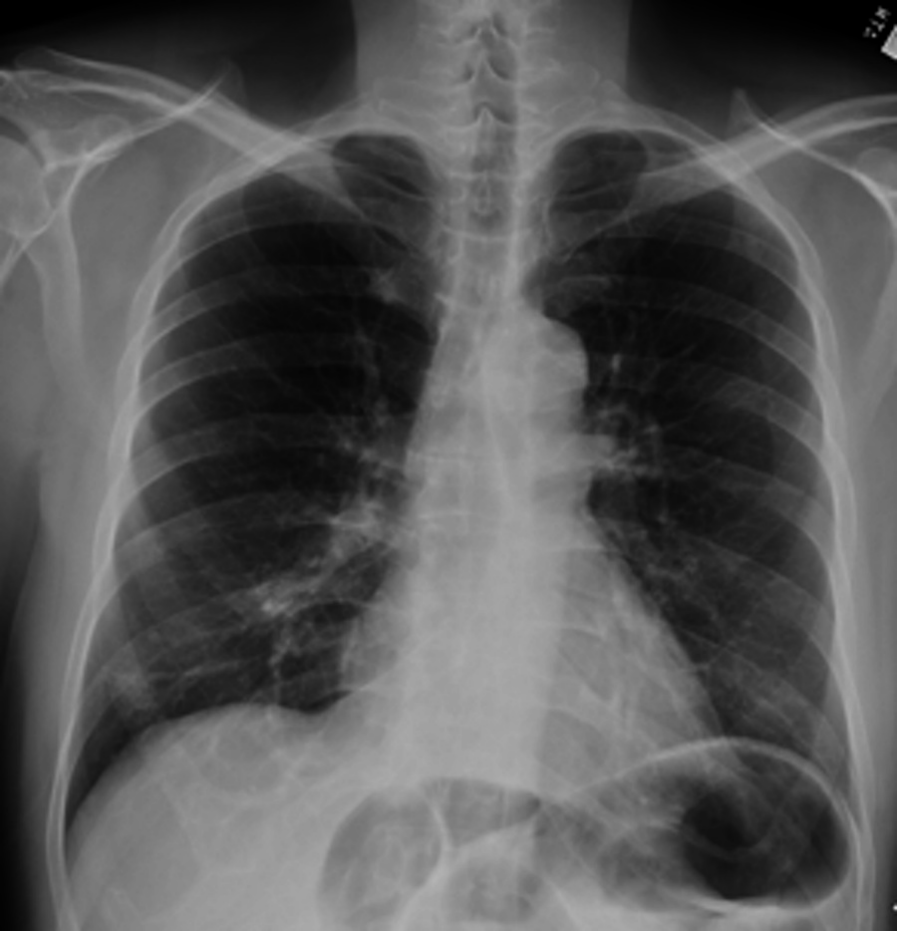
Chest X-ray taken eight months later showed significant resolution of the nodules/masses.

A CT-guided core needle biopsy from the left posterior pelvic mass that was noted six months after the initial presentation showed high grade diffuse large B-cell lymphoma involving skeletal muscles. The pathology report showed diffuse proliferation of lymphocytes, which were positive for cluster of differentiation (CD) 20 and B-cell lymphoma (BCL)-2 by immunohistochemistry tests. He was started on chemotherapy with rituximab, cyclophosphamide, doxorubicin, vincristine, and prednisone (R-CHOP) with subsequent complete clinical and radiologic remission (Figure 6).

## Discussion

BALToma is rarely seen in a healthy adult; however, it has been observed with increased prevalence among patients with prior bronchitis-bronchiolitis and bronchiectasis. This correlation with chronic inflammatory condition is remarkably similar to gastric mucosa-associated lymphoid tissue (MALT) lymphoma, which suggests that they too may be caused by the chronic inflammation induced by a microbiologic agent [4]. We know that *helicobacter pylori* is known to be the causative organism in gastric MALToma. Although the coexistence of BALToma with tuberculosis has been reported in several studies [5], at present no microbiologic agent has been confirmed as the causative organism for pulmonary MALToma. Similar to observations noted in other cases, the fact that our patient developed MALToma while being treated for latent tuberculosis might highlight the possible association between the two.

Patients with BALTomas usually have an indolent clinical course. Most patients are asymptomatic and when present, the symptoms are non-specific and include dyspnea, cough, fever, hemoptysis and weight loss. B-symptoms are uncommon in MALToma, and herald the development of other complications like diffuse large B-cell lymphoma. A study conducted by Jung, et al. revealed that 61.5% of patients had no pulmonary or constitutional symptoms [6].

The radiographic appearance of primary pulmonary lymphoma is variable. The lesions can appear as airspace consolidation or nodule(s) and can be multiple and involve both lungs [7].

Diagnosis can be made by obtaining tissue specimens—either by open thoracotomy or video-assisted thoracoscopic surgery. Specific diagnosis can be made using cell marker study or molecular techniques such as flow cytometry [8]. In our cases, however, those studies were not performed. However, it is recommended to rule out other differentials including reactive changes with biopsy.

For the management of MALT lymphoma, initial observation can be a reasonable option for asymptomatic patients with indolent clinical course [6]. However, for a patient who has symptoms, single-agent or combination chemotherapy can be considered. In the study by Jung, et al. either single-agent chlorambucil or combination therapy with cyclophosphamide, vincristine, and prednisone (CVP) was given to eight patients with MALT lymphoma. Five patients achieved complete resolution while three achieved partial resolution [6]. Similarly, our patient was symptomatic and received R-CHOP treatment with complete remission.

With regards to treatment options for non-MALT lymphoma, which are mostly intermediate to high grade tumors with worse prognosis, adjuvant chemotherapy or radiation therapy need to be considered [9].

The other treatment option for localized disease is the use of radiation therapy. Localized MALT lymphomas have excellent prognosis following moderate-dose radiation therapy. In one study, among patients who were treated for localized MALToma with radiation, a complete response was noted in 66/69 patients [10]. The overall survival for patients with MALT lymphoma was superior to those with non-MALT lymphoma and the disease rarely transforms into secondary diffuse large B-cell lymphoma (DLBCL).

## Conclusions

We recommend that BALTomas be considered in patients with chronic pulmonary symptoms and previous history of pulmonary tuberculosis especially in those showing suboptimal clinical response to usual antibiotic treatment. A tissue diagnosis is indicated in cases presenting with consolidations that do not respond to appropriate therapy. A lung biopsy with adequate tissue sample for immunological studies is indicated for accurate diagnosis. We also recommend further research into establishing an etiologic association between prior tuberculous exposure and the development of MALToma so that a more effective strategic management can be drawn.
